# Testing a decoy donation incentive to improve online survey participation: Evidence from a field experiment

**DOI:** 10.1371/journal.pone.0299711

**Published:** 2024-02-29

**Authors:** Sandro Tiziano Stoffel, Biswajit Chaki, Ivo Vlaev

**Affiliations:** 1 Research Department of Behavioural Science and Health, UCL, London, United Kingdom; 2 Institute of Pharmaceutical Medicine, University of Basel, Basel, Switzerland; 3 Warwick Business School, University of Warwick, Coventry, United Kingdom; The Ohio State University College of Medicine and Public Health: The Ohio State University College of Medicine, UNITED STATES

## Abstract

This study introduces a new randomized field experiment exploring the impact of offering a decoy charity donation incentive together with a monetary reward to increase response rates in an online survey about coronavirus fears. The study used a two-stage approach, starting with a preliminary survey to investigate participant attitudes toward different types of donations. Subsequently, an experiment was conducted wherein a less desirable £2 donation (the decoy) was introduced as an alternative to a £2 Amazon voucher (the target) within the choice set. The study sample consisted of 431 university students. They were split into three groups: a control group with a standard £2 Amazon voucher incentive (216 participants), a decoy group with the target shown first (108 participants), and a decoy group with the decoy shown first (107 participants). We found significantly higher survey completion rates in the decoy than in the control condition (82.3% vs. 74.5%). Notably, an order effect was observed–presenting the target before the decoy led to a higher completion rate (89.8%) compared to presenting the decoy first (74.8%). Importantly, the inclusion of the decoy incentive did not introduce any response bias. This study offers a proof of principle that incorporating a decoy charity donation incentive into the choice set can have a positive impact on survey participation without adversely affecting response behaviour. It demonstrates the potential of such incentives to encourage participants to complete online surveys, even when a small monetary reward is offered.

## Introduction

Health surveys are vital for grasping population health and intervention efficacy [1]. Recent research highlights declining online survey responses, which impact research validity [2]. Enhancing response rates is thus a priority [3]. Various factors affect online survey response rates, including questionnaire length, incentive, structure, persuasion, difficulty, communication, participant interests, and sender identity [3–8]. Recent systematic reviews have shown that monetary incentives can increase involvement in surveys [9,10]. Small monetary incentives can increase participation [10], especially prepaid incentives because of psychological obligation [11,12]. The reciprocity principle explains participation as a result of perceiving incentives as reciprocal gifts [13], while economic exchange theory explains it with anticipated rewards [14].

While non-monetary incentives like charitable donations can boost participation [15], people generally favour monetary rewards over non-monetary ones [16–20]. Despite some studies presuming charity’s suitability [21–23], it hasn’t consistently improved response rates [19,24–26].

A recent study demonstrated that offering a decoy survey with less convenient questions and delayed remuneration increases survey participation [27]. The decoy effect, also known as the asymmetric decoy effect or attraction effect, enhances preferences for an option (the target) by introducing a less attractive alternative (the decoy) [28], representing a well-studied context-dependent preference enhancement [29]. This effect operates by making the target appear more favourable in comparison to the inferior decoy, thus increasing the probability of selecting the more attractive target [28]. While extensively explored in various contexts [30,31], the decoy effect’s role in survey participation is limited [27]. This study extends that research by examining decoy incentives, specifically their impact on survey completion when a donation incentive is provided. It is hypothesized that including the decoy incentive increases the attractiveness and likelihood of selecting the standard incentive (the target).

## Methodology

### Study design

The study design followed a previous study by Stoffel and colleagues [27] and contained two stages. In the first stage (preliminary study), participants were recruited for a brief survey to collect email addresses, and to assess their attitudes towards various incentives (e.g., Amazon vouchers and donations to different charities). In the second stage (experiment), the participants who provided their email addresses were sent the invite to fill out the main questionnaire. The data collection for both the preliminary and final survey was conducted in July 2023. Study participants were recruited from a UK-based university through different social media platforms like WhatsApp, Facebook, WeChat as well as official email groups of different departments of the University. In line with the previous study, participants were eligible to participate in the study if they were an undergraduate, postgraduate, or Ph.D. student at the University of Warwick. We further excluded 3 study participants from the experiment due to their age, as they were significantly older than others.

### Preliminary survey

The preliminary survey questionnaire was intended to recruit students from the University of Warwick to assess their eligibility and preference for different incentives (see S1 Text in the supplementary file). The invitation stated that study participants were needed for a survey on the fear of the coronavirus. Interested individuals were invited to click on the survey link for registration. The information sheet did not disclose the experimental nature of the study. After providing consent to participate in the study, interested participants were asked to provide an email address to receive the main survey and respond to some demographic questions. Additionally, they were asked to indicate their choice for the most preferred and least favoured incentive as participation fee. The specific incentives being assessed included a £2 Amazon voucher as well as donation of the £2 participation fee to four potential charities like (1) *Donkey Sanctuary*, (2) *University of Warwick*, *Horses and Ponies Protection Association*, (3) *Veterinarian for Animal Welfare Zimbabwe* (UK), (4) *World Association for Transport Animal Welfare and Studies*. The four charities were chosen as they allowed individual donations of £2.

### Experiment

Eligible participants who provided their email addresses in the preliminary survey were individually randomised into the control and decoy conditions. Additionally, to explore the order effect, respondents in the decoy condition were further randomized into two sub-groups at a 1:1 ratio: 1) decoy shown first, and 2) target shown first. Invitations for the main survey were sent a week after the preliminary survey and no reminders were employed. The invitations urged study participation to complete the brief questionnaire within 7 days. Survey completers of the experiment received their incentives the following week.

### Questionnaire in the control condition

Participants in the control condition received a standard survey invitation email (see S1 Fig). They were informed of a £2 Amazon voucher incentive upon survey completion. The control questionnaire comprised demographic questions on age, gender, ethnicity, and education level and the eight close-ended questions of the validated Fear of the Coronavirus Questionnaire (FCQ, [32]). The survey concluded with an attention check question and a debrief question about reasons for participation (see S2 Text in the supplementary file). To measure survey completion, none of the questions were mandatory.

### Questionnaire in the decoy condition

Participants in the decoy condition received an email containing two distinct URL links: one for the standard £2 Amazon voucher incentive (target) and another for the decoy incentive. The order of presentation for these links was randomized. Both options were displayed in tabular format within the email to facilitate easy comparison (see S2 and S3 Figs). The survey for the decoy condition was identical to the one in the control condition, with an extra debrief question about the preferred incentive: Amazon voucher, donation, or no preference (see S3 Text in the supplementary file).

### Outcomes

The primary outcome was the proportion of individuals selecting the target incentive and completing the survey across the two experimental conditions. Consistent with prior research [26,32], the single participant who opted for the decoy and completed the questionnaire was categorized as not having selected the target. Additionally, we investigated whether the decoy introduction influenced data quality by investigating the attention question and the FCQ scores. Non-response bias was assessed by comparing the socio-demographic characteristics of respondents who completed the questionnaire across the two experimental conditions [33].

### Preregistration and ethics approval

Ethics approval for the present study was received from the Humanities and Social Science Research Ethics Committee (HSSREC) and all data files and materials are publicly available via the Open Science Framework at https://osf.io/qundv/. Written informed consent was obtained from all study participants prior to their involvement in the research using tick boxes.

### Statistical analyses

The required sample size for the experiment was determined pre-data collection, assuming around 40% control and 50% decoy completion rates [27]. With a minimum of 180 participants per condition, the experiment had 80% power and an alpha value of 0.05 to detect such a difference [34]. The primary outcome was assessed with a Chi-square test of independence and multivariate logistic regressions, adjusting for demographics. The FCQ scores between the control and decoy groups were compared using a Student t-test. Statistical analysis was conducted using Stata/IC version 16.0 (StataCorp LP, College Station, TX).

## Results

### Preliminary survey

**Fig 1:**
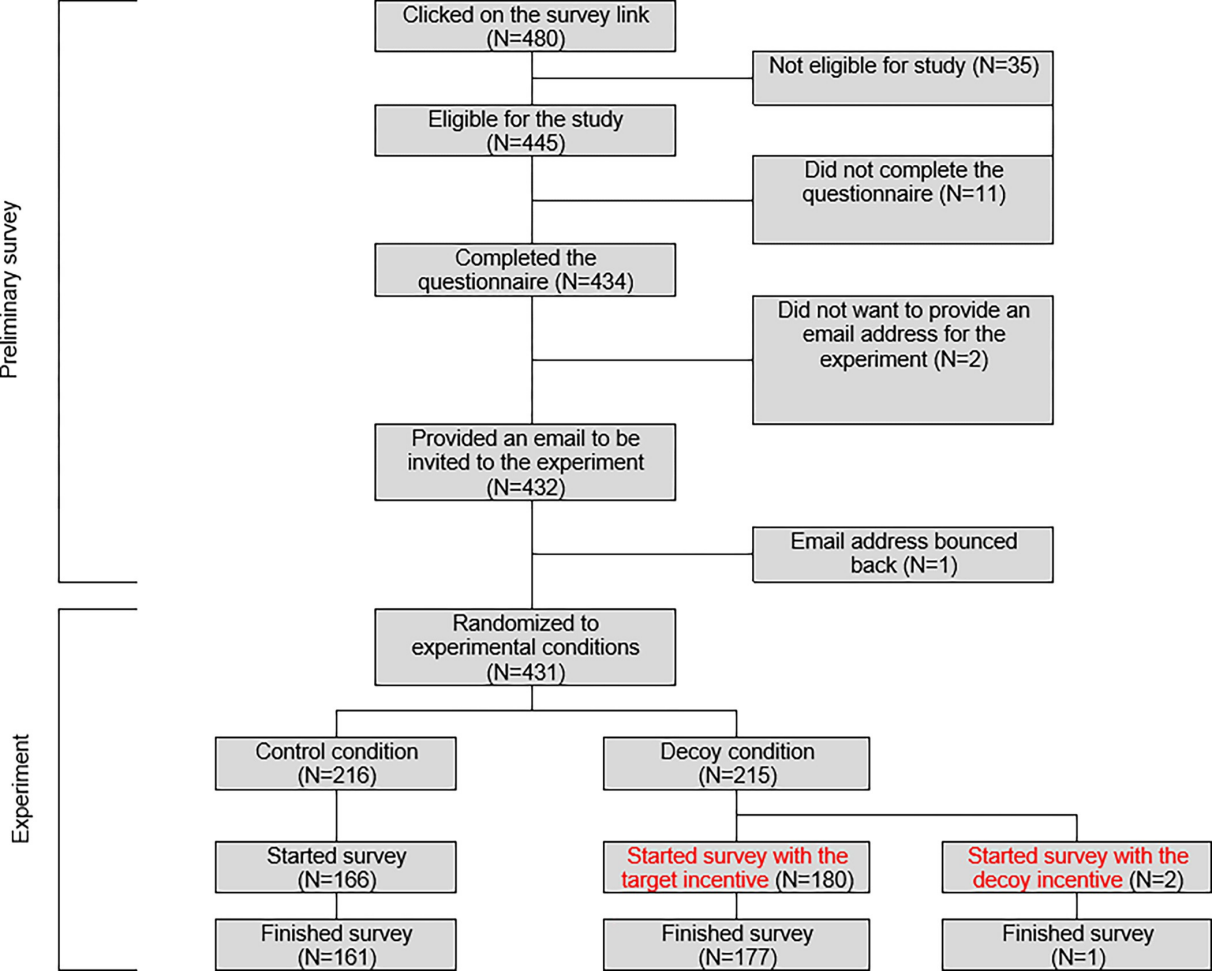
Flow through the study.

Study sample. 480 participants started the preliminary survey. Of these, 11 didn’t complete it, 35 were ineligible, and 2 chose not to share emails, resulting in a sample size of 432 (refer to Fig 1). A majority were female (78%, 337/432), aged 22–25 (47.9%, 207/432), held mixed ethnicity (30.3%, 131/432), and had some university education without a degree (46.8%, 202/432; see S1 Table).

**Fig 2:**
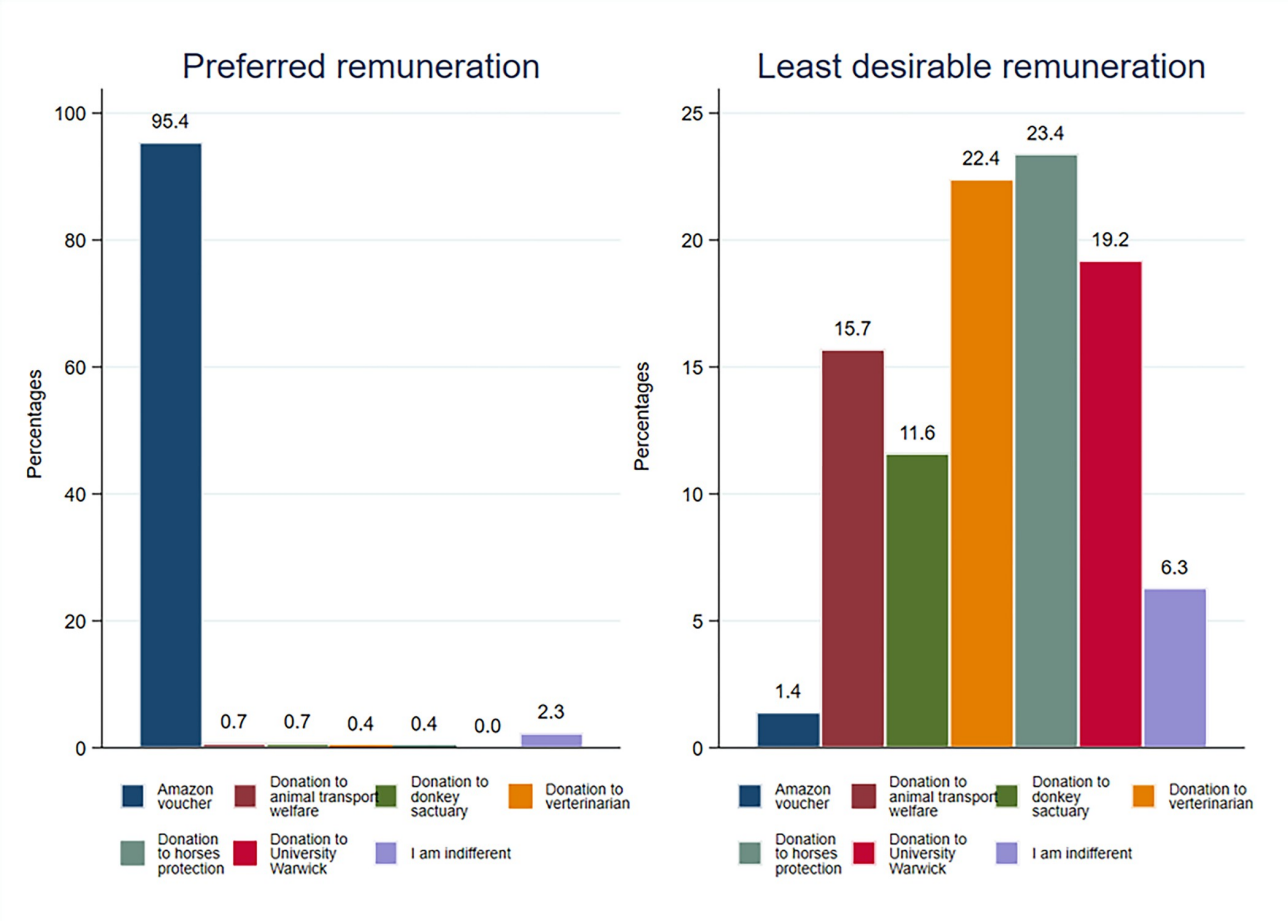
Stated preference for remuneration in the preliminary survey (N = 432).

Attitude towards the incentives. Fig 2 illustrates that 95.4% of participants (412/432) favoured the Amazon voucher as their preferred remuneration. Conversely, *The Horses and Ponies Protection Association* was the least favoured incentive, disliked by 23.4% (101/432), followed by *Veterinarian for Animal Welfare Zimbabwe (UK)* (22.4%; 97/432) and *University of Warwick* (19.2%; 82/432). For administrative simplicity and compliance with university regulations, the experiment used the University of Warwick charity.

### Experiment

Study sample. Of 432 emails sent to participants from the preliminary survey, 1 bounced back, resulting in a sample of 431 (216 in control, 215 in decoy). Sociodemographic variables aligned with the preliminary survey (refer to S2 Table).

**Fig 3:**
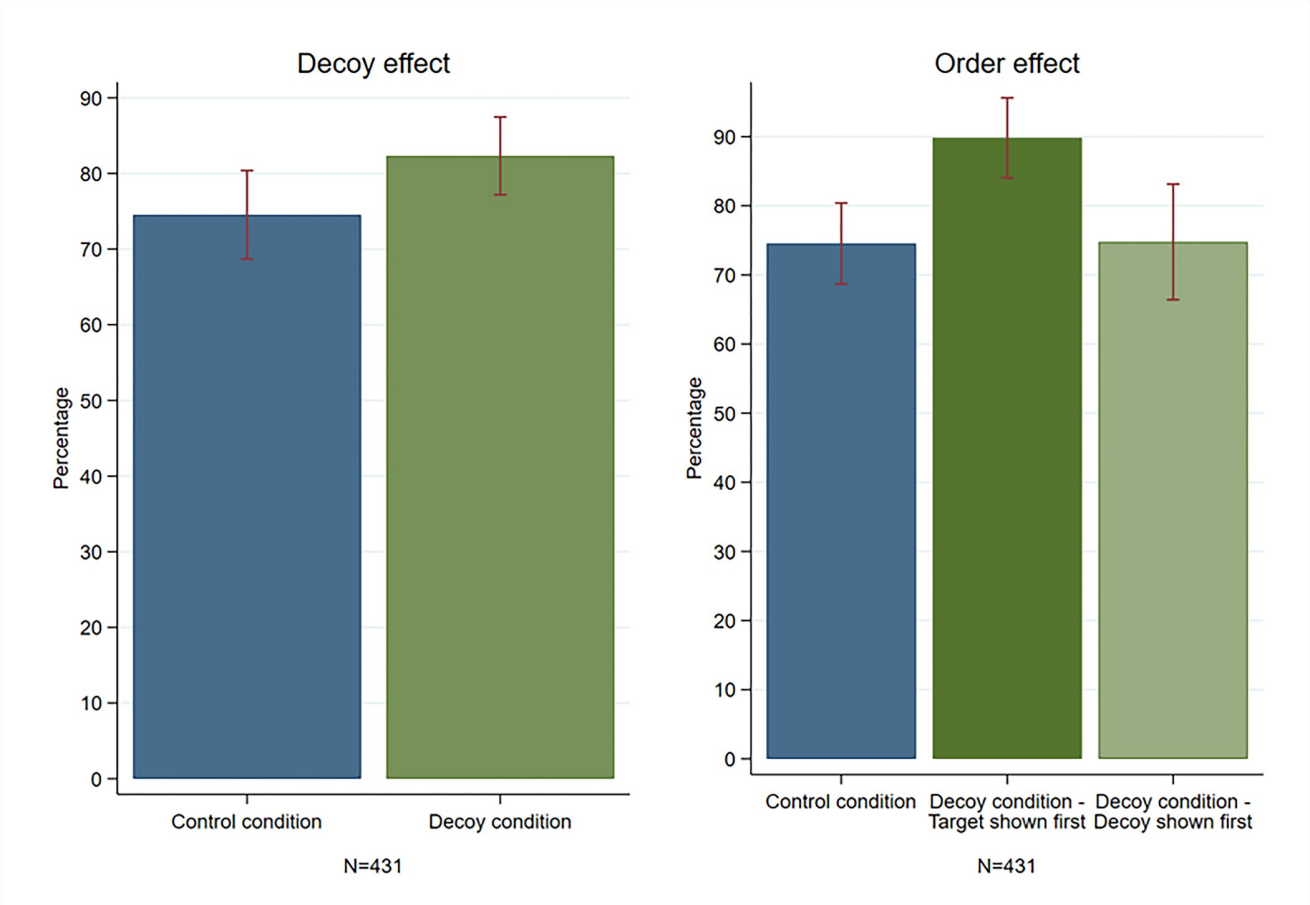
Participation in the survey with the target incentive across the experimental.

Effect on survey participation. In Fig 3, more completed the survey with the target incentive in the decoy condition than in the control condition (82.3% vs. 74.5%, χ2(1, N = 431) = 3.86, p = 0.049). Binary logistic regressions in Table 1 indicate non-significance in the unadjusted model (OR 1.59, 95% CI: 0.999–2.534, p = 0.050), but significance in the fully adjusted model (aOR 1.98, 95% CI: 1.164–3.356, p = 0.012).

**Table 1:** Binary logistic regression on completing the survey with the target incentive (N = 431).

		Unadjusted model		Adjusted model	
	(%)	OR	95% CI	aOR	95% CI
**Overall**	(78.4)				
**Condition**					
Control	(74.5)	Ref.		Ref.	
Decoy	(82.3)	1.591	0.999–2.534	1.977	1.164–3.356*
**Age**					
18–21 years old	(67.5)	Ref.		Ref.	
22–25 years old	(78.2)	1.720	0.963–3.073	1.318	0.694–2.503
26–30 years old	(84.5)	2.613	1.361–5.016**	2.394	1.100–5.212*
**Gender**					
Male	(68.8)	Ref.		Ref.	
Female	(81.3)	1.964	1.171–3.294*	0.727	0.404–1.309
Non-binary	(50.0)	0.453	0.027–7.499	0.534	0.028–10.312
**Ethnicity**					
White	(67.3)	Ref.		Ref.	
Asian or Asian British	(76.5)	1.582	0.869–2.879	1.391	0.711–2.721
Mixed	(75.6)	1.501	0.844–2.671	1.969	1.049–3.694*
Black, Arab or other	(98.8)	40.279	5.370–302.156**	33.332	3.808–291.794**
**Education**					
Some University education but no degree	(63.9)	Ref.		Ref.	
Bachelor’s Degree	(94.2)	9.266	4.293–20.000**	11.153	4.772–26.068**
Graduate degree or prefer not to say	(86.7)	3.678	1.878–7.204**	1.035	0.412–2.602
N		431		431	

* *p*<0.05

** *p*<0.01.

Order effect. Fig 3 indicates that survey completion significantly increased when the target incentive was presented before the decoy incentive (89.8% vs. 74.5%, aOR 4.17; 95% CI: 1.962–8.883, p<0.001, refer to Table 2). However, displaying the decoy before the target incentive showed no decoy effect (74.8% vs. 74.5%, aOR 1.10; 95% CI: 0.599–2.049, p = 0.745).

**Table 2:** Binary logistic regression on completing the survey with the target incentive looking at the order of the presentation (N = 431).

		Unadjusted model		Adjusted model	
	(%)	OR	95% CI	aOR	95% CI
**Overall**	(78.4)				
**Condition**					
Control	(74.5)	Ref.		Ref.	
Decoy–Target first	(89.8)	3.012	1.504–6.034**	4.175	1.962–8.883**
Decoy–Decoy first	(74.8)	1.012	0.594–1.725	1.108	0.599–2.049
**Age**					
18–21 years old	(67.5)	Ref.		Ref.	
22–25 years old	(78.2)	1.720	0.963–3.073	1.328	0.689–2.560
26–30 years old	(84.5)	2.613	1.361–5.016**	2.458	1.115–5.419*
**Gender**					
Male	(68.8)	Ref.		Ref.	
Female	(81.3)	1.964	1.171–3.294*	0.761	0.418–1.384
Non-binary	(50.0)	0.453	0.027–7.499	0.600	0.031–11.676
**Ethnicity**					
White	(67.2)	Ref.		Ref.	
Asian or Asian British	(76.5)	1.582	0.869–2.879	1.385	0.698–2.748
Mixed	(75.6)	1.501	0.844–2.671	2.028	1.067–3.855*
Black, Arab or other	(98.8)	40.279	5.370–302.156**	37.789	4.248–336.139**
**Education**					
Some University education but no degree	(63.9)	Ref.		Ref.	
Bachelor’s Degree	(94.2)	9.266	4.293–20.000**	11.704	4.982–27.495**
Graduate degree or prefer not to say	(85.7)	3.678	1.878–7.204**	0.938	0.364–2.414
N		431		431	

* *p*<0.05

** *p*<0.01.

Effect on FCQ scale and non**-**response bias. The analysis of sociodemographic variables and FCQ responses reveals no differences among those who completed the survey in both conditions showed no disparities (see S3 Table and S4 Fig). Mean FCQ scores were comparable between control (23.73) and decoy conditions (23.65, t(336) = 0.06, p = 0.565; see S3 Table). While comparisons between those who completed the survey and those who did not reveal that mainly individuals aged 26–30, women, of Black or Black British or Arab ethnicity, or those with a Bachelor’s degree completed the survey, the decoy incentive did not seem to create this non-response bias, as the pattern can be observed within the two experimental conditions (see S4–S6 Tables).

Attention check and debrief questions. All survey completers correctly answered the attention question, and a majority (55.9%, 189/338) stated incentives as their primary motivation for participation. Additionally, 22.2% (75/338) participated to support research, while 21.9% (74/338) were interested in the survey topic. Of those motivated by incentives, a majority (89.4%, 169/189) preferred the £2 Amazon vouchers, while the rest (20.6%, 20/189) were indifferent to the incentives.

## Discussion

This study presents a proof of principle that pairing a decoy charity donation incentive with a monetary reward can increase survey participation. In line with previous research, the preliminary survey showed that most study participants prefer a monetary incentive over charitable donations [16,25]. The experiment unveiled a significantly decoy effect in that including a donation option in the choice set increased the likelihood that individuals chose and complete the survey with the target incentive. Similar to Stoffel and colleagues [27], we find a distinct order effect, in that only presenting the monetary incentive first, followed by the decoy donation, heightened survey completion rates compared to reversed presentation. This is in line with another recent study that found the decoy effect is influenced by the framing of the decoy alternative [35]. In our experiment, the order effect exhibits a primacy bias, as individuals tend to consider the option listed first rather than last [36]. Studies have shown that simply changing the order of the alternatives can influence decision-making [37].

Furthermore, similar as in previous research, the decoy incentive’s inclusion showed no response bias or impact on survey responses [27]. Nonetheless, differences exist in the baseline completion rates in the two experimental conditions, which were significantly higher in this study. Additionally, the magnitude of the decoy effect observed here was notably smaller. While this divergence could stem from the nature of the decoy alternative—we varied the incentive, our study’s decoy was also dominated in only one attribute, compared to Stoffel et al.’s [27] two attributes. Previous research has shown that the decoy effect depends on how strongly the decoy is dominated by the target [38]. Additionally, our decoy incentive wasn’t ranked least desirable, leaving room for a stronger effect had the least desirable option been employed.

We found that mainly individuals aged 26–30, females, of Black or Black British or Arab ethnicity, or those with a Bachelor’s degree completed the survey with the target incentive. While studies suggest that the decoy effect emerges at a young age and that testosterone is associated with inconsistent decisions [39,40], our study does not indicate that male participants were more likely to react to the decoy. Our study presents limitations that warrant further investigation. Firstly, the analytical sample was comprised of university students, which may be more sensitive to incentives, limiting so the possibility to generalize the findings to the public. Additionally, while donations to one’s own institution has been found to be an ineffective incentive [23], it is plausible that different welfare-oriented charities (e.g., medical research) could generate varied decoy effects. Moreover, the study involved a degree of deception by informing participants of a coronavirus fear study. Future research could employ a comprehensive design to explore the decoy effect without deceptive practices and a two-stage design, thus reducing the risk of priming and reducing the baseline completion rates.

## Conclusion

This study is the first investigation to explore how offering a decoy charity donation incentive for a survey can improve response rate of a survey providing monetary incentive. Unlike prior research comparing charity donations and monetary rewards, our study demonstrates how charity donations effectively serve as decoys to boost survey engagement. Beyond the decoy effect, our research highlights an order effect: presenting the monetary reward before the decoy incentive significantly increases survey completion rates. Importantly, the decoy incentive doesn’t seem to introduce bias or alter responses. While the decoy effect may be modest, this research underscores the effectiveness of a decoy donation in encouraging survey participation, even with minimal financial incentives. This study furthers understanding of respondent behaviour in incentive-driven surveys and invites nuanced exploration of testing decoy effects.

## Supporting information

S1 Fig Invitation email for the control condition. (DOCX)

S2 Fig Invitation email for the decoy condition-target shown first. (DOCX)

S3 Fig Invitation email for the decoy condition-decoy shown first. (DOCX)

S4 Fig Distribution of FCQ score across the two experimental conditions (N = 338). (DOCX)

S1 Table Description of the study sample in the preliminary survey (N = 432). (DOCX)

S2 Table Description of the study sample in the experiment (main survey) (N = 431). (DOCX)

S3 Table Characteristics of individuals choosing the survey with the target incentive (N = 338). (DOCX)

S4 Table Overall non-response bias (N = 431). (DOCX)

S5 Table Non-response bias in control condition (N = 216). (DOCX)

S6 Table Non-response bias in decoy condition (N = 215). (DOCX)

S1 Text Preliminary survey questionnaire. (DOCX)

S2 Text Survey used in main experiment (control condition). (DOCX)

S3 Text Survey used in main experiment (decoy condition). (DOCX)
