# Supplementary figures and images for: Testing a decoy donation incentive to improve online survey participation: Evidence from a field experiment
Source: PLoS One. 2024 Feb 29;19(2):e0299711. doi: 10.1371/journal.pone.0299711 (PMC10903882; doi:10.1371/journal.pone.0299711)

**Figure S1:** Invitation email for the control condition


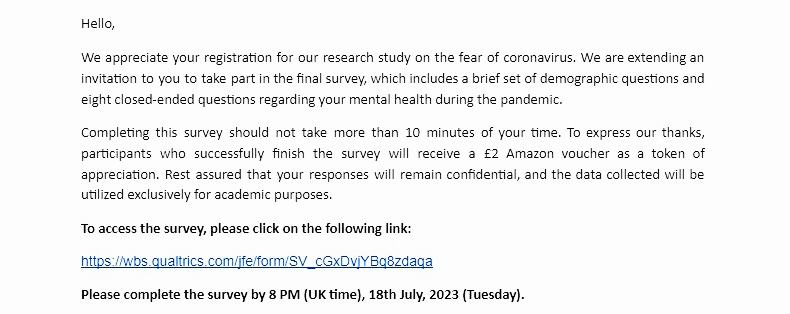

Supplement: S1 Fig — (DOCX) [file pone.0299711.s001.docx]

**Figure S2:** Invitation email for the decoy condition-target shown first


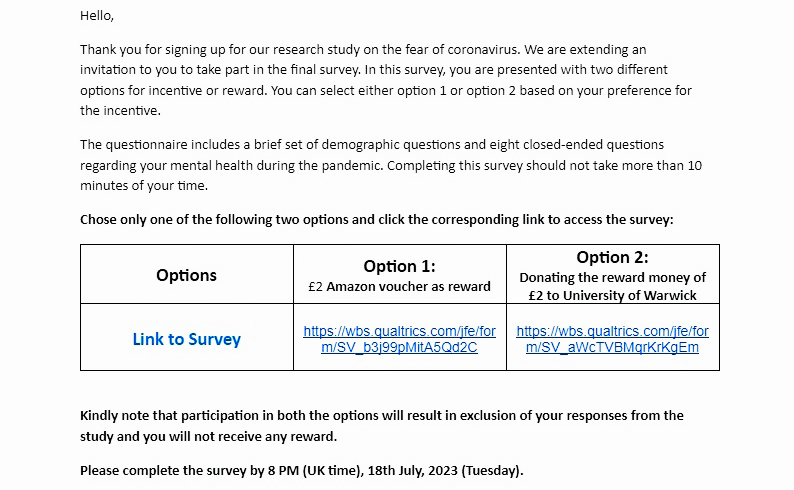

Supplement: S2 Fig — (DOCX) [file pone.0299711.s002.docx]

**Figure S3:** Invitation email for the decoy condition-decoy shown first


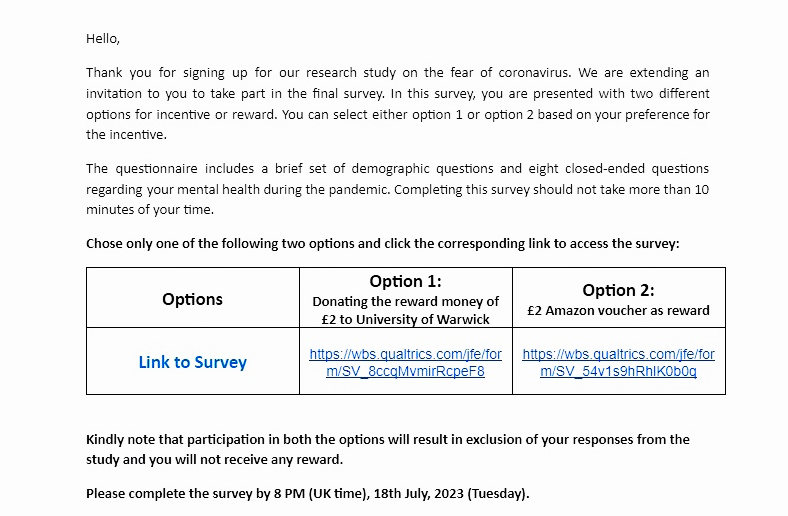

Supplement: S3 Fig — (DOCX) [file pone.0299711.s003.docx]

**Figure S4: Distribution of FCQ Score across the two experimental conditions (N=338)**


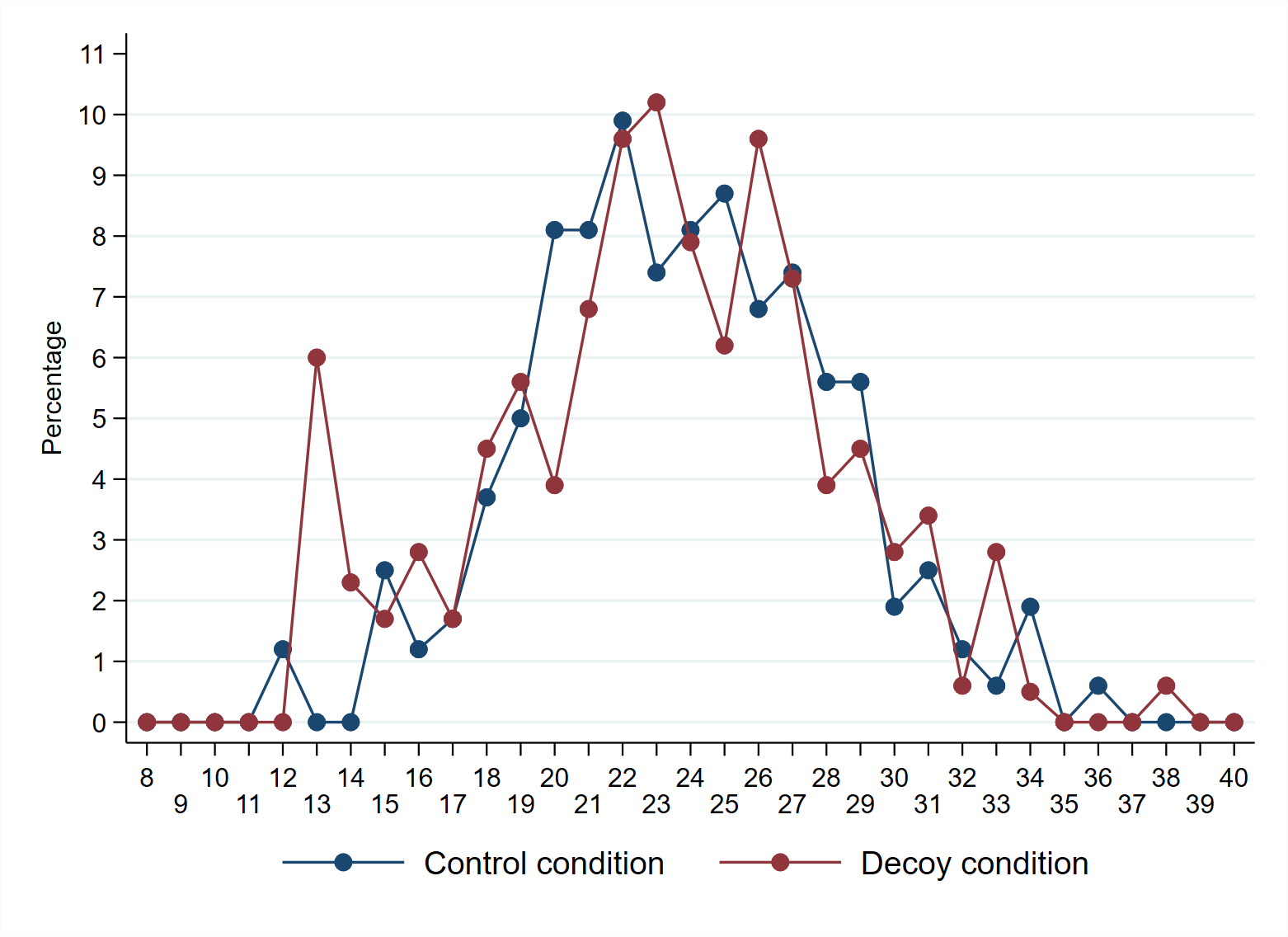

Supplement: S4 Fig — (DOCX) [file pone.0299711.s004.docx]
