# Supplementary material for: Testing a decoy donation incentive to improve online survey participation: Evidence from a field experiment
Source: PLoS One. 2024 Feb 29;19(2):e0299711. doi: 10.1371/journal.pone.0299711 (PMC10903882; doi:10.1371/journal.pone.0299711)
Supplement: S2 Table — (DOCX) [file pone.0299711.s006.docx]

**Table S2. Description of the study sample in the experiment (main survey) (N=431)**

|  | | N | (%) |
| --- | --- | --- | --- |
| Age | |  |  |
|  | 18-21 years old | 77 | (17.9) |
|  | 22-25 years old | 206 | (47.8) |
|  | 26-30 years old | 148 | (34.3) |
| Gender | |  |  |
|  | Male | 93 | (21.6) |
|  | Female | 336 | (77.9) |
|  | Non-binary | 2 | (0.5) |
| Ethnicity | |  |  |
|  | White | 101 | (23.4) |
|  | Asian or Asian British | 115 | (26.7) |
|  | Mixed | 131 | (30.4) |
|  | Black or Black British | 40 | (9.3) |
|  | Arab | 23 | (5.3) |
|  | Other or unknown | 21 | (4.9) |
| Education Level | |  |  |
|  | Some University education but no degree | 202 | (46.9) |
|  | Bachelor’s Degree | 139 | (32.3) |
|  | Graduate or professional degree | 67 | (15.5) |
|  | Prefer not to say | 23 | (5.3) |
