# Supplementary material for: Testing a decoy donation incentive to improve online survey participation: Evidence from a field experiment
Source: PLoS One. 2024 Feb 29;19(2):e0299711. doi: 10.1371/journal.pone.0299711 (PMC10903882; doi:10.1371/journal.pone.0299711)
Supplement: S3 Table — (DOCX) [file pone.0299711.s007.docx]

**Table S3. Characteristics of individuals choosing the survey with the target incentive (N=338)**

|  |  | Control condition (N=161) | | Decoy condition  (N=177) | | p-value* | Total  (N=338) | |
| --- | --- | --- | --- | --- | --- | --- | --- | --- |
|  | | N | (%) | N | (%) |  | N | (%) |
| Age | | |  |  |  |  |  |  |
|  | 18-21 years old | 25 | (15.5) | 27 | (15.2) | 0.931 | 52 | (15.4) |
|  | 22-25 years old | 75 | (46.6) | 86 | (48.6) |  | 161 | (47.6) |
|  | 26-30 years old | 61 | (37.9) | 64 | (36.2) |  | 125 | (37.0) |
| Gender | | |  |  |  |  |  |  |
|  | Male | 32 | (19.9) | 32 | (18.1) | 0.626^+^ | 64 | (18.9) |
|  | Female | 128 | (79.5) | 145 | (81.9) |  | 273 | (80.8) |
|  | Non-binary | 1 | (0.6) | 0 | (0.0) |  | 1 | (0.3) |
| Ethnicity | | |  |  |  |  |  |  |
|  | White | 32 | (19.9) | 36 | (20.3) | 0.799 | 68 | (20.1) |
|  | Asian or Asian British | 42 | (26.1) | 46 | (26.0) |  | 88 | (26.0) |
|  | Mixed | 48 | (29.8) | 51 | (28.8) |  | 99 | (29.3) |
|  | Black or Black British | 22 | (13.7) | 18 | (10.2) |  | 40 | (11.8) |
|  | Arab | 10 | (6.2) | 13 | (7.3) |  | 23 | (6.8) |
|  | Other or unknown | 7 | (4.3) | 13 | (7.3) |  | 20 | (5.9) |
| Education | | |  |  |  |  |  |  |
|  | Some University education but no degree | 55 | (34.2) | 74 | (41.8) | 0.272 | 129 | (38.2) |
|  | Bachelor’s Degree | 71 | (44.1) | 60 | (33.9) |  | 131 | (38.8) |
|  | Graduate or professional degree | 24 | (14.9) | 31 | (17.5) |  | 55 | (16.3) |
|  | Prefer not to say | 11 | (6.8) | 12 | (6.8) |  | 23 | (6.8) |
| CFQ score - Mean and SD | | 23.73 | [4.46] | 23.65 | [4.68] | 0.565^o^ | 23.69 | [4.57] |

* Chi-Square goodness of fit

^+^ Fisher’s exact test

^o^ Student’s T-test
