# Supplementary material for: Testing a decoy donation incentive to improve online survey participation: Evidence from a field experiment
Source: PLoS One. 2024 Feb 29;19(2):e0299711. doi: 10.1371/journal.pone.0299711 (PMC10903882; doi:10.1371/journal.pone.0299711)
Supplement: S4 Table — (DOCX) [file pone.0299711.s008.docx]

**Table S4. Overall non-response bias (N=431)**

|  | | Participated | | Did not participate | | p-value* |
| --- | --- | --- | --- | --- | --- | --- |
|  | |  |  | N | (%) |  |
| Age | |  | |  | |  |
|  | 18-21 years old | 52 | (15.4) | 25 | (26.9) | 0.014 |
|  | 22-25 years old | 161 | (47.6) | 45 | (48.4) |  |
|  | 26-30 years old | 125 | (37.0) | 23 | (24.7) |  |
| Gender | |  | |  | |  |
|  | Male | 64 | (18.9) | 29 | (31.2) | 0.013+ |
|  | Female | 273 | (80.8) | 63 | (67.7) |  |
|  | Non-binary | 1 | (0.3) | 1 | (1.1) |  |
| Ethnicity | |  | |  | |  |
|  | White | 68 | (20.1) | 33 | (35.5) | <0.001+ |
|  | Asian or Asian British | 88 | (26.0) | 27 | (29.0) |  |
|  | Mixed | 99 | (29.3) | 32 | (34.4) |  |
|  | Black or Black British | 40 | (11.8) | 0 | (0.0) |  |
|  | Arab | 23 | (6.8) | 0 | (0.0) |  |
|  | Other or unknown | 20 | (5.9) | 1 | (1.1) |  |
| Education | |  | |  | |  |
|  | Some University education but no degree | 129 | (38.2) | 73 | (78.5) | <0.001+ |
|  | Bachelor’s Degree | 131 | (38.8) | 8 | (8.6) |  |
|  | Graduate or professional degree | 55 | (16.3) | 12 | (12.9) |  |
|  | Prefer not to say | 23 | (6.8) | 0 | (0.0) |  |

* Chi-Square goodness of fit

^+^ Fisher’s exact test
