# Supplementary material for: Testing a decoy donation incentive to improve online survey participation: Evidence from a field experiment
Source: PLoS One. 2024 Feb 29;19(2):e0299711. doi: 10.1371/journal.pone.0299711 (PMC10903882; doi:10.1371/journal.pone.0299711)
Supplement: S5 Table — (DOCX) [file pone.0299711.s009.docx]

**Table S5. Non-response bias in control condition (N=216)**

|  | | Participated | | | Did not participate | | | p-value* |
| --- | --- | --- | --- | --- | --- | --- | --- | --- |
|  | | N | | (%) | N | | (%) |  |
| Age | | | | |  | | |  |
|  | 18-21 years old | 25 | (15.5) | | 11 | (20.0) | | 0.731 |
|  | 22-25 years old | 75 | (46.6) | | 25 | (45.5) | |  |
|  | 26-30 years old | 61 | (37.9) | | 19 | (34.5) | |  |
| Gender | | | | |  | | |  |
|  | Male | 32 | (19.9) | | 17 | (30.9) | | 0.134+^+^ |
|  | Female | 128 | (79.5) | | 37 | (67.3) | |  |
|  | Non-binary | 1 | (0.6) | | 1 | (1.8) | |  |
| Ethnicity | | | | |  | | |  |
|  | White | 32 | (19.9) | | 19 | (34.6) | | 0.002+ |
|  | Asian or Asian British | 42 | (26.1) | | 17 | (30.9) | |  |
|  | Mixed | 48 | (29.8) | | 18 | (32.7) | |  |
|  | Black or Black British | 22 | (13.7) | | 0 | (0.0) | |  |
|  | Arab | 10 | (6.2) | | 0 | (0.0) | |  |
|  | Other or unknown | 7 | (4.3) | | 1 | (1.8) | |  |
| Education | | | | |  | | |  |
|  | Some University education but no degree | 55 | (34.2) | | 44 | (80.0) | | <0.001+ |
|  | Bachelor’s Degree | 71 | (44.1) | | 3 | (5.5) | |  |
|  | Graduate or professional degree | 24 | (14.9) | | 8 | (14.5) | |  |
|  | Prefer not to say | 11 | (6.8) | | 0 | (0.0) | |  |

* Chi-Square goodness of fit

^+^ Fisher’s exact test
