# Supplementary material for: Testing a decoy donation incentive to improve online survey participation: Evidence from a field experiment
Source: PLoS One. 2024 Feb 29;19(2):e0299711. doi: 10.1371/journal.pone.0299711 (PMC10903882; doi:10.1371/journal.pone.0299711)
Supplement: S6 Table — (DOCX) [file pone.0299711.s010.docx]

**Table S6. Non-response bias in decoy condition (N=215)**

|  | | Participated | | Did not participate | | | p-value* |
| --- | --- | --- | --- | --- | --- | --- | --- |
|  | | N | (%) | N | (%) | |  |
| Age | |  | |  | | |  |
|  | 18-21 years old | 27 | (15.2) | 14 | | (36.8) | 0.001+ |
|  | 22-25 years old | 86 | (48.6) | 20 | | (52.6) |  |
|  | 26-30 years old | 64 | (36.2) | 4 | | (10.5) |  |
| Gender | |  | |  | | |  |
|  | Male | 32 | (18.1) | 12 | | (31.6) | 0.076+^+^ |
|  | Female | 145 | (81.9) | 26 | | (68.4) |  |
|  | Non-binary | 0 | (0.0) | 0 | | (0.0) |  |
| Ethnicity | |  | |  | | |  |
|  | White | 36 | (20.3) | 14 | | (36.8) | 0.012+ |
|  | Asian or Asian British | 46 | (26.0) | 10 | | (26.3) |  |
|  | Mixed | 51 | (28.8) | 14 | | (36.8) |  |
|  | Black or Black British | 18 | (10.2) | 0 | | (0.0) |  |
|  | Arab | 13 | (7.3) | 0 | | (0.0) |  |
|  | Other or unknown | 13 | (7.3) | 0 | | (0.0) |  |
| Education | |  | |  | | |  |
|  | Some University education but no degree | 74 | (41.8) | 29 | | (76.3) | 0.001+ |
|  | Bachelor’s Degree | 60 | (33.9) | 5 | | (13.2) |  |
|  | Graduate or professional degree | 31 | (17.5) | 4 | | (10.5) |  |
|  | Prefer not to say | 12 | (6.8) | 0 | | (0.0) |  |

* Chi-Square goodness of fit

^+^ Fisher’s exact test
