# Supplementary material for: Testing a decoy donation incentive to improve online survey participation: Evidence from a field experiment
Source: PLoS One. 2024 Feb 29;19(2):e0299711. doi: 10.1371/journal.pone.0299711 (PMC10903882; doi:10.1371/journal.pone.0299711)
Supplement: S1 Text — (DOCX) [file pone.0299711.s011.docx]

**Text S1. Preliminary Survey Questionnaire**

*Collect Email Address*

Q1. This questionnaire is to recruit participants. The main questionnaire will be sent via email in one week. Would you like to provide your email address for further study?

- Yes
- No

Q2. What is your mailing address?

*Demographic Questions*

Q1. What is your age group?

- Below 18 years
- 18-21 years old
- 22-25 years old
- 26-30 years old
- Above 30 years

Q2. What gender do you identify as?

- Male
- Female
- A gender identity not listed here.
- Prefer not to say.

Q3. Which of these best describe your ethnic group?

- White
- Mixed
- Asian or Asian British
- Black or Black British
- Arab
- Other
- Prefer not to say.

Q4. What is your current education level?

- Some University education but no degree
- University - bachelor’s degree
- Graduate or professional degree (MA, MS, MBA, PhD, Law Degree, Medical Degree etc_
- Prefer not to say

Q5. At which university do you study?

- University of Warwick
- Other UK university
- …..

*Preference Questions*

Q1. When you are invited to fill in a questionnaire, which type of incentive would you prefer the most?

- £ 2 Amazon voucher as reward
- Donating the reward of £ 2 to Donkey Sanctuary
- Donating the reward of £ 2 to University of Warwick
- Donating the reward of £ 2 to Horses and Ponies Protection Association
- Donating the reward of £ 2 to Veterinarians for Animal Welfare Zimbabwe (UK)
- Donating the reward of £ 2 to World Association for Transport Animal Welfare and Studies.
- I am indifferent between the above options.

Q2. When invited to participate in a survey, which of the following incentives would you prefer the least?

- £ 2 Amazon voucher as reward
- Donating the reward of £ 2 to Donkey Sanctuary
- Donating the reward of £ 2 to University of Warwick
- Donating the reward of £ 2 to Horses and Ponies Protection Association
- Donating the reward of £ 2 to Veterinarians for Animal Welfare Zimbabwe (UK)
- Donating the reward of £ 2 to World Association for Transport Animal Welfare and Studies.
- I am indifferent between the above options.
