# Supplementary material for: Testing a decoy donation incentive to improve online survey participation: Evidence from a field experiment
Source: PLoS One. 2024 Feb 29;19(2):e0299711. doi: 10.1371/journal.pone.0299711 (PMC10903882; doi:10.1371/journal.pone.0299711)
Supplement: S3 Text — (DOCX) [file pone.0299711.s013.docx]

**Text S3. Survey used in main experiment (decoy condition)**

What is your e-mail address?

*Demographic Questions*

Q1. What is your age group?

- Below 18 years
- 18-21 years old
- 22-25 years old
- 26-30 years old
- Above 30 years

Q2. What gender do you identify as?

- Male
- Female
- A gender identity not listed here.
- Prefer not to say

Q3. Which of these best describe your ethnic group?

- White
- Mixed
- Asian or Asian British
- Black or Black British
- Arab
- Other
- Prefer not to say

Q4. What is your current education level?

- Some University education but no degree
- University - bachelor’s degree
- Graduate or professional degree (MA, MS, MBA, PhD, Law Degree, Medical Degree etc_
- Prefer not to say

Q5. At which university do you study?

- University of Warwick
- Other UK university
- …..

*Fear of Coronavirus Questionnaire*

Q1. Please select the extent to which the following thoughts, feelings and behaviors apply to you: (anchors: 1 = “Strongly disagree”; 5 = “Strongly agree”)


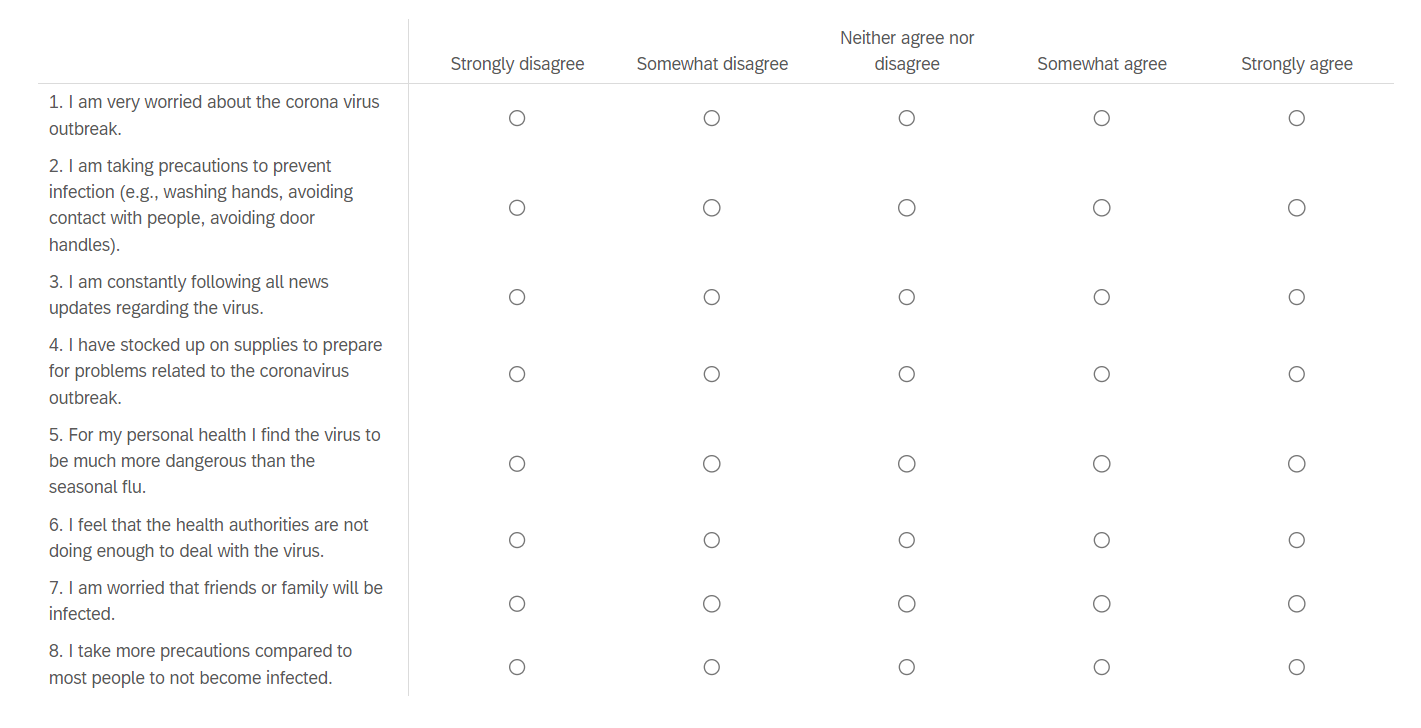


*Attention question.*

Please select “disagree” to show that you are paying attention to this question.

- Strongly agree
- Agree
- Disagree
- Strongly disagree

*Debrief Questions*

Q1 What is the main reason to respond to the survey?

- Personal interest in the survey topic
- Incentives associated with participating.
- Contribution to research and data collection
- Other Reason (Please specify)

Q2. Which incentive was more attractive to you?

- £2 Amazon voucher as reward
- Donating the reward of £2 to University of Warwick
- Both were equally attractive
